# Docking-based inverse virtual screening: methods, applications, and challenges

**DOI:** 10.1007/s41048-017-0045-8

**Published:** 2018-02-01

**Authors:** Xianjin Xu, Marshal Huang, Xiaoqin Zou

**Affiliations:** 1 Dalton Cardiovascular Research Center, University of Missouri, Columbia, MO 65211 USA; 2 Department of Physics and Astronomy, University of Missouri, Columbia, MO 65211 USA; 3 Informatics Institute, University of Missouri, Columbia, MO 65211 USA; 4 Department of Biochemistry, University of Missouri, Columbia, MO 65211 USA

**Keywords:** Inverse virtual screening, Target fishing, Polypharmacology, Side effects, Drug repositioning, Molecular docking

## Abstract

Identifying potential protein targets for a small-compound ligand query is crucial to the process of drug development. However, there are tens of thousands of proteins in human alone, and it is almost impossible to scan all the existing proteins for a query ligand using current experimental methods. Recently, a computational technology called docking-based inverse virtual screening (IVS) has attracted much attention. In docking-based IVS, a panel of proteins is screened by a molecular docking program to identify potential targets for a query ligand. Ever since the first paper describing a docking-based IVS program was published about a decade ago, the approach has been gradually improved and utilized for a variety of purposes in the field of drug discovery. In this article, the methods employed in docking-based IVS are reviewed in detail, including target databases, docking engines, and scoring function methodologies. Several web servers developed for non-expert users are also reviewed. Then, a number of applications are presented according to different research purposes, such as target identification, side effects/toxicity, drug repositioning, drug–target network development, and receptor design. The review concludes by discussing the challenges that docking-based IVS needs to overcome to become a robust tool for pharmaceutical engineering.

## Introduction

Identifying protein targets for a query ligand is a crucial aspect of drug discovery. Historically, natural products derived from plants, animals, micro-organisms, *etc*., were used as medicines to cure many diseases. The accumulated experience and knowledge of their usages have become an abundant resource for modern drug discovery (Ji *et al.* 2009). Although purified compounds from these natural products present good therapeutic activities, molecular mechanisms of action including the identification of binding targets are often shrouded in mystery. The drug design process in modern times is highly dependent on Ehrlich’s assumption (Kaufmann 2008), in which drugs work as “magic bullets” modulating one target of particular relevance to a disease. Great success has been achieved with this simple assumption, while disadvantages are also emerging in recent years. The most visible disadvantage is the high attrition rate (about 90%) of potential compounds at the late stage of clinical trials due to certain efficacy and clinical safety problems (Nwaka and Hudson 2006). A number of drugs have been withdrawn from the market because of serious side effects or life-threatening toxicities. Recent studies also suggest that each existing drug binds to, on average, about six target proteins instead of one (Azzaoui *et al.* 2007; Mestres *et al.* 2008). If all the targets of an interested ligand can be identified at the early stage of new drug design, the side effects and toxicities that appear in the later stages of clinical trials can be effectively avoided. Thus, a prescreening process can significantly increase the success rate and reduce the development cost for the overall drug pipeline. However, the lack of effective experimental tools in identifying all the potential targets for a small molecule on a proteome-wide scale remains a daunting challenge to overcome.

**Fig. 1:**
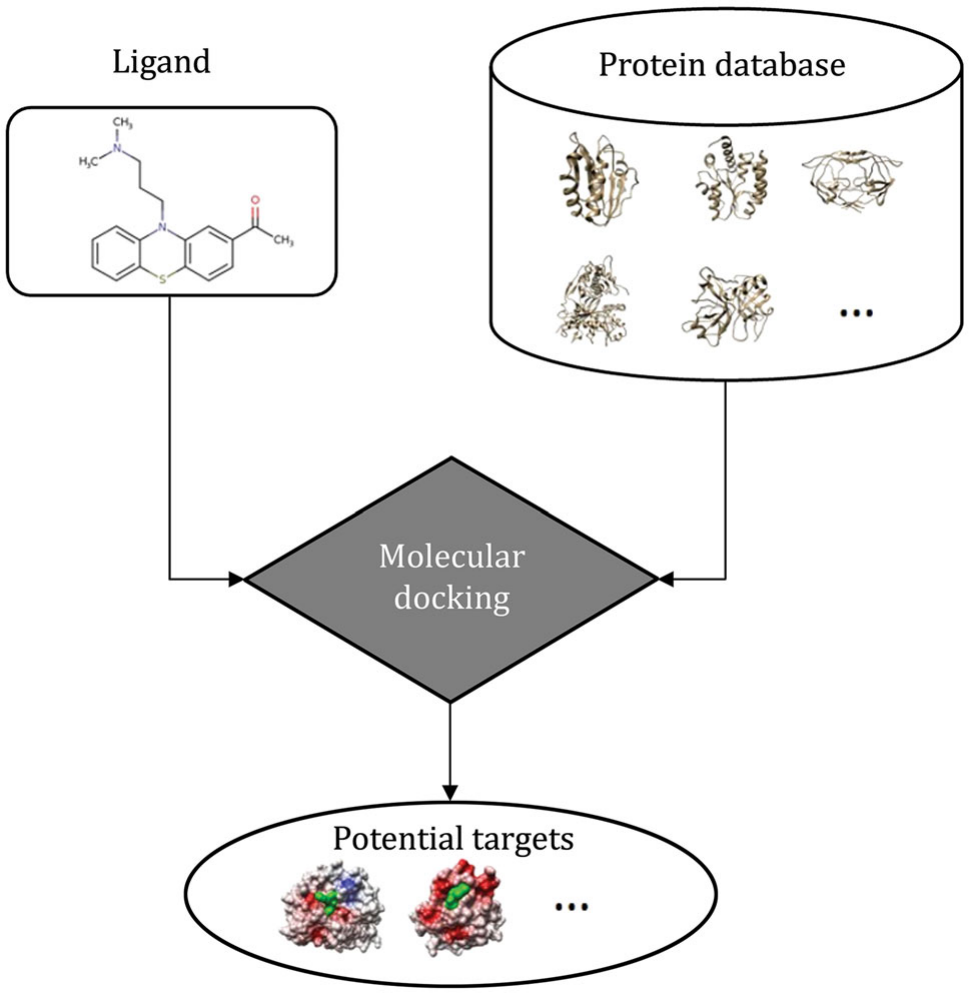
A flowchart of the docking-based inverse virtual screening

Recently, an inverse virtual screening (IVS) technology based on molecular docking methods has been developed and widely used for the process of target identification (Chen and Zhi 2001). A molecular docking method is defined as the prediction of both the binding mode and binding affinity of a query ligand (such as a small-molecule drug) against a receptor (such as a target protein) (Brooijmans and Kuntz 2003; Sousa *et al.* 2006; Grinter and Zou 2014a, b). In the IVS method, a molecular docking process is employed to screen a protein database for a query ligand, and then an enriched subset containing possible targets of the ligand is provided. Figure 1 shows a flowchart of the docking-based IVS procedure.

To run a docking-based IVS study, at least two components are required, a protein database and a molecular docking program. The target database is a collection of structures of proteins or active sites. With the rapidly increasing number of structures deposited in the Protein Data Bank (PDB) (Berman *et al.* 2000), a desirable target database can be constructed for docking-based IVS. The target database can also be extended through homology modeling techniques. Then, a potentially interesting small molecule is docked to each element of the target database by a docking program. Generally, a docking program consists of two main components—the sampling algorithm and the scoring function. The sampling component generates sufficient putative binding modes. The scoring function further ranks these modes based on binding energy evaluations. The ability of the existing scoring functions to accurately predict binding energies remains limited (Brooijmans and Kuntz 2003; Huang *et al.* 2010). Fortunately, the purpose of IVS studies (and of virtual screening of potent ligands against a query target) is in pursuit of an enriched subset of potential candidates (*e.g.*, top 1% of the ranked proteins in the IVS case or top 1% of the ranked ligands in the virtual screening case), which is a relatively less challenging task than binding energy prediction for a scoring function.

In addition to docking-based IVS, there are several other computational methods that can be used for target identification, including ligand-based methods, binding site comparisons, protein–ligand interaction fingerprints, and so on (Rognan 2010; Koutsoukas *et al.* 2011; Xie *et al.* 2011; Ma *et al.* 2013). Ligand-based methods are based on the molecular similarity principle, which states that molecules with similar structures tend to have similar biological activities (Willett *et al.* 1998; Bender and Glen 2004). These methods heavily rely on the pre-existing knowledge about the molecules in the database, and require a database of small molecules with known binding targets. Although ligand-based methods are widely used for target identification and have achieved a great amount of success, they become utterly useless for the remaining “unknown space” (*i.e.*, dissimilar ligands). Similarly, for the methods of binding site comparison and protein–ligand interaction fingerprinting, at least one protein–ligand complex structure of the query small molecule is required (Rognan 2010). All the aforementioned approaches are classified as “knowledge-based” IVS methods. By contrast, docking-based IVS is the only method that does not rely on such preliminary information, rendering it a more attractive option in the field of target identification.

Ever since the first docking-based IVS program was developed by Chen *et al.* (Chen and Zhi 2001), the method has been improved and utilized widely for various purposes in the field of drug discovery. Here, we review the method of docking-based IVS, including the target database, docking engine, and scoring function components of this method. We also review the web servers that integrate the complex process of IVS for non-expert users. Then, we present published studies in which docking-based IVS played an important role. These application studies are classified into target identification, side effect/toxicity assessments, drug repositioning, multi-target therapy/drug–target network, and receptor design. Finally, we discuss about current challenges that docking-based IVS needs to overcome in order to become a robust tool for far-reaching applications.

## Docking-based IVS

In docking-based IVS, a given small molecule is docked to the binding site of each protein in a target database through a docking engine. Then, target proteins are ranked according to the binding scores estimated by a scoring function. This complex process has been integrated and presented as online web servers for non-expert utilization. These components are explained in detail as follows.

### Target databases

A database consisting of three-dimensional protein structures is required for the implementation of docking-based IVS. Owing to the development of technologies in structural biology, such as X-ray crystallography and NMR spectroscopy, an increasing number of protein crystal structures have been resolved and deposited in a publicly accessible database, the PDB (Berman *et al.* 2000). Up to the present (16th March 2017), the number of protein entries in the PDB has reached up to 118,663, which provides an abundant resource for constructing a sub-database for IVS.

For example, screening-PDB (sc-PDB) (Kellenberger *et al.* 2006) is a sub-database extracted from the PDB for the purpose of virtual screening. sc-PDB collects all the high-resolution crystal structures of protein–ligand complexes in which ligands are nucleotides (<4-mer), peptides (<9-mer), cofactors, and organic compounds. In the latest version v.2013, sc-PDB contains 9283 entries corresponding to 3678 different proteins and 5608 different ligands. The known protein–ligand complex structures in the database embed the information about the binding sites (*i.e.*, the pocket where the ligand binds), which would significantly reduce the sampling space for docking. The authors’ indiscriminate collections enrich the sc-PDB database, but also complicate the subsequent analysis of the screening results. To address this issue, several databases that focus on specific topics have been constructed, and are introduced as follows.

Therapeutic target database (TTD) (Chen *et al.* 2002) focuses on known and potential therapeutic targets, which are proteins and nucleic acids collected from literature. Important information, such as targeted diseases, pathway information, and corresponding drugs/ligands, is provided in the database. After the latest update in 2015 (Yang *et al.* 2016), TTD contains 2589 targets, including 397 successful, 723 clinical trial, and 1469 research targets. However, the TTD database does not provide 3D structures of the targets, which need to be downloaded from the PDB database by users.

Potential drug–target database (PDTD) (Gao *et al.* 2008) is another database focusing on therapeutic targets. Different to TTD, PDTD contains only protein targets. Impressively, cleaned 3D structures for both protein and active sites are provided, minimizing the complexity of docking preparation for users. After the latest update in 2008, PDTD contains 1207 entries, covering 841 known and potential drug targets. Targets in the PDTD database were further categorized into several subsets according to two criteria: therapeutic areas and biochemical criteria. These subsets could be very effective for studies on a special topic. The database was implemented in an online web server TarFisDock (Li *et al.* 2006), which will be introduced later in this review.

Drug adverse reaction database (DART) (Ji *et al.* 2003) focuses on known and potential targets corresponding to the adverse effects of drugs. Information such as physiological function, binding affinity of known ligands, and corresponding adverse effects is provided. Currently, the DART database contains entries for 147 ADR targets and 89 potential targets. The structures of the targets and the active sites in the database need to be prepared by users.

Recently, our group presented a small molecule-transcription factor (SM-TF) database containing all the targetable TFs with known 3D structures (Xu *et al.* 2016). SM-TF contains 934 entries, covering 176 TFs from a variety of species. Besides the protein structures, the co-bound ligands are also provided in the SM-TF database. Therefore, the database is suitable for both docking-based IVS and ligand-based IVS.

In addition to the aforementioned freely accessible databases, researchers often construct highly specialized datasets. For example, a dataset containing enzymes was constructed by Macchiarulo *et al.* to study the selectivity and competition of metabolites between enzymes (Macchiarulo *et al.* 2004). Zahler *et al.* collected a dataset of protein kinase structures for identifying the targets of kinase inhibitors (Zahler *et al.* 2007). Lauro *et al.* (2011) collected a dataset of proteins involved in cancer and tumor development for antitumor target identification of natural bioactive compounds. These individualized datasets can be either directly derived from a protein–ligand complex structure database like sc-PDB, or constructed by collecting information from publically accessible drug–target databases such as SuperTarget (Günther *et al.* 2008), BindingDB (Liu *et al.* 2007), and DrugBank (Wishart *et al.* 2006), as listed in Table 1. It should be noted that information in the later databases is redundant. The 3D structures of proteins need to be downloaded from the PDB database by users, and further preparations are necessary to fit the input file format of docking methods.

**Table 1:** Publicly available databases containing the information about targetable proteins

Database	Description	URL
PDB	A pool of 3D structures of macromolecules, including proteins, nucleic acids, and complex assemblies. The total number of structures deposited in the database is more than 12,000	http://www.rcsb.org
sc-PDB	A subset of PDB with the collection of protein–ligand complexes. In the latest version v.2013, the database contains 9283 entries corresponding to 3678 different proteins and 5608 different ligands	http://bioinfo-pharma.u-strasbg.fr/scPDB
TTD	Therapeutic target database (TTD) contains 2360 targets, including 2589 targets, including 397 successful, 723 clinical trial, and 1469 research targets	http://bidd.nus.edu.sg/group/ttd
PDTD	Potential drug–target database (PDTD) contains 1207 entries covering 841 known and potential drug targets, which can be further categorized into subsets according to two criteria: therapeutic areas and biochemical criteria. Structures for both protein and active site are available	http://www.dddc.ac.cn/pdtd
DART	Drug adverse reaction database (DART) contains 147 ADR targets and 89 potential targets	http://bidd.nus.edu.sg/group/drt
SM-TF	A database of 3D structures of small molecule-transcription factor complexes. The database contains 934 entries, covering 176 TFs from a variety of species	http://zoulab.dalton.missouri.edu/SM-TF
SuperTarget	A database contains the information about drug–target relations. The database contains >6000 target proteins, 196,000 compounds, 282 drug–target-related pathways, and >6000 drug–target-related ontologies	http://bioinformatics.charite.de/supertarget
BindingDB	A database of measured binding affinities for drug–targets with small, drug-like molecules. Until now, the database contains more than 1,000,000 binding data, for about 7997 protein targets and 453,657 small molecules	http://www.bindingdb.org/bind
DrugBank	In the latest version (5.0), the database contains 8261 drug entries including 2021 FDA-approved small-molecule drugs, 233 FDA-approved biotech (protein/peptide) drugs, 94 nutraceuticals, and over 6000 experimental drugs. 4338 non-redundant protein sequences are linked to these drug entries	http://www.drugbank.ca

Some of them can be directly used for docking-based IVS studies. Others are abundant resources for constructing an individualized target dataset

### Docking engines

Prediction of protein–ligand complex structures plays an essential role in docking-based IVS. The credibility of predicted binding patterns of a ligand against each protein target is crucial to the final success. Fortunately, plenty of programs have been developed for the purpose of structure prediction of protein–ligand complexes (Brooijmans and Kuntz 2003; Sousa *et al.* 2006). Here, we focus on the issues closely related to IVS. Interested readers are referred to other recent reviews on molecular docking methods for more information (Brooijmans and Kuntz 2003; Sousa *et al.* 2006; Huang and Zou 2010; Grinter and Zou 2014a, b).

Briefly, a molecular docking program is designed to predict a complex structure based on the known 3D structures of its components. In other words, a docking method is a problem of searching for the ligand location on a given protein target (referred to as binding site prediction) and then for the ligand conformations and orientations in the binding site. Although methods of global blind docking are provided by most docking programs, they suffer from time-consuming execution and a low success rate compared to dockings into a known binding site. Considering the large number of proteins in the target database, protein structures with known active sites are preferred in the preparation of a target database.

In the early stages of the development of the docking methods, both the ligand and the receptor were treated rigidly. A shape matching method was employed to place a ligand in the binding site of a receptor. Only six degrees of freedom (three translational and three rotational) of a ligand conformation are considered, which is computationally efficient. However, binding of a ligand to a receptor is a mutual fitting progress, with conformational changes in both components. Thus, conformational search is necessary for both the ligand and the receptor during docking.

According to the searching method, ligand flexibility algorithms can be divided into three types: systematic, stochastic, and deterministic search. Systematic search generates all possible ligand binding conformations by exploring the whole conformational space. Despite the completeness of sampling, the number of evaluations increases rapidly as the number of degrees of freedom are increased (*i.e.*, the number of rotatable bonds in a ligand). Examples of systematic search include exhaustive search implemented in Glide (Friesner *et al.* 2004), and a fragmentation method named incremental construction algorithm implemented in LUDI (Bohm 1992) and DOCK (DesJarlais *et al.* 1986). Stochastic algorithms sample the ligand conformational space by making random changes, which will be accepted or rejected according to a probabilistic criterion. This type of methods significantly reduces computational efforts for large systems; however, the uncertainty of convergence is a major concern. Examples of stochastic algorithms are Monte Carlo (MC) methods implemented in MCDOCK (Liu and Wang 1999), and evolutionary algorithms implemented in GOLD (Jones *et al.* 1997) and AutoDock (Morris *et al.* 1998). For deterministic search, the final state of the system depends on the initial state. Examples are energy minimization methods and molecular dynamics (MD) simulations. Systems are thus guided to states with lower energies. However, it is difficult to cross energy barriers, and systems are often trapped in local minima with these methods.

The flexibility of the receptor remains a big challenge for docking, because of the huge number of degrees of freedom in the system. Some methods for ligand flexibility are also applicable for receptor flexibility, such as the aforementioned evolutionary algorithms, MC, and MD methods. In addition, several approaches accounted for partial flexibility within the receptor, such as soft docking and conformer libraries. Soft docking allows an overlap between the ligand and the receptor by softening the interatomic van der Waals (vdW) interactions (Jiang and Kim 1991). The methods based on conformer libraries can be further divided into two different types. The first type describes the side-chain conformations by a rotamer library and keeps the backbones fixed (Leach 1994). The second type is referred to docking with multiple receptor structures, using pre-generated receptor conformers (Knegtel *et al.* 1997). Other methods, such as induced fit docking (IFD), change both protein and ligand conformations to fit each other during the docking process (Sherman *et al.* 2006). Theoretically, these methods can account for receptor flexibility in terms of either the side chains or the backbones, or both. However, the rapidly growing degrees of freedom make even a single docking event very time-consuming, and make the hopes of implementing IVS a mirage.

According to a recent review that exhaustively presented the programs available for protein–ligand docking, the number of available docking programs was more than 50 and kept increasing (Sousa *et al.* 2013). It is difficult to say which docking program is better than the others, because the performance of most docking programs is highly dependent on the system of study, *e.g.*, the characteristics of both the receptor and the ligand (Sousa *et al.* 2013). In the published literature related to docking-based IVS, the choice of a docking engine is quite arbitrary.

### Scoring functions

The scoring function is another important component of protein–ligand docking protocols. It is for evaluation and ranking of the binding conformations generated by the searching algorithms described in the last section. In fact, scoring functions are usually implemented in docking programs. Here, we artificially separate scoring functions from docking engines, not only because scoring functions play an essential role in every docking protocol, but also because they are employed to pick potential targets out of a database in IVS.

Scoring functions for molecular docking can be grouped into three major classes according to how they are derived: force field-based, empirical, and knowledge-based. Parameters in force field-based scoring functions are derived from molecular mechanical force fields used in MD simulations, including contributions from vdW interactions, electrostatic interactions, and bond stretching/bending/torsional potentials. The desolvation effects can be considered by using implicit solvent models like the Poisson–Boltzmann/surface area (PB/SA) model (Baker *et al.* 2001; Grant *et al.* 2001; Rocchia *et al.* 2002) and the generalized-Born/surface area (GB/SA) model (Still *et al.* 1990; Hawkins *et al.* 1995; Qiu *et al.* 1997). However, the solvent models would significantly slow down the computational speed, which must be considered in screening studies. In addition, the absence of entropic terms is also a weakness of this type of scoring functions. For example, force-based scoring functions are used in docking programs such as DOCK (Meng *et al.* 1992) and GOLD (Jones *et al.* 1997). The second kind of scoring functions are empirical scoring functions, which are a sum of different energy terms such as vdW, electrostatics, hydrogen bond, desolvation, entropy, hydrophobicity, and so on. The weight of each energy term is generated based on a training set of experimental affinity data. The empirical scoring functions are easy to calculate and take much less computational time than force-filed-based scoring functions. However, the accuracy of an empirical scoring function heavily relies on the training set of experimental affinity data. Examples can be found in docking programs such as FlexX (Rarey *et al.* 1996), Glide (Friesner *et al.* 2004), ICM (Abagyan *et al.* 1994), and LUDI (Bohm 1994, 1998). The third kind of scoring functions are knowledge-based, which are also known as statistical potential-based scoring functions. They are developed by statistical analysis of the atom pair occurrence frequencies in a training set of experimentally determined protein–ligand complex structures. Briefly summarized, the frequency of structural features (such as atom pairs) that appear in a training dataset is used to derive the scoring functions. The relationship between the frequency of the structural features and the interaction energies assigned to those features relies on the inverse-Boltzmann equation (Thomas and Dill 1996). Compared to the previous two types of scoring functions, knowledge-based scoring functions hold a good balance between accuracy and speed. However, a weakness of knowledge-based scoring functions is that it is still training set-dependent. Examples of knowledge-based scoring functions are potential of mean force (PMF) (Muegge and Martin 1999; Muegge 2006) and ITScore (Huang and Zou 2006a, b; Grinter *et al.* 2013; Grinter and Zou 2014a, b; Yan *et al.* 2016). The interested reader is recommended to read recent reviews on scoring functions for protein–ligand docking (Huang *et al.* 2010; Grinter and Zou 2014a, b).

Generally, the best (*i.e.*, the lowest) docking score from each protein–ligand docking is used for ranking the proteins in the database. Proteins with low docking scores are potential targets for the ligand. Then, proteins among the top 1% (or 5%) of the ranking list can be used for further analysis. However, this arbitrary cutoff results in enormous false positive targets, significantly increasing the degree of difficulty. Meanwhile, some real targets beyond the cutoff will be ignored. Although false positives and false negatives remain an open question in IVS, several efforts have been made to reduce false positive and false negative targets in the final predicted list.

In a pioneer work of docking-based IVS by Chen *et al.* (Chen and Zhi 2001), an energy threshold was introduced to filter the proteins in the ranking list. The method was based on an analysis of the known protein–ligand complexes in the PDB, which showed that the computed protein–ligand interaction energy was generally less than $\Delta E_{\text{Threshold}} = -\alpha N\;{\text{kcal}}/{\text{mol}}$. Here, *N* is the number of ligand atoms, and $\alpha$ is a constant (~1.0) which can be determined by fitting the equation for a large set of PDB structures. Proteins with calculated binding energies less than $\Delta E_{\text{Threshold}}$ were predicted as potential targets. Furthermore, to consider competitive binding against natural ligands *in vivo*, another energy threshold, $\Delta E_{\text{Competitor}}$, was introduced. $\Delta E_{\text{Competitor}}$ is the binding energy of a competitive natural ligand interacting with each protein for a query ligand. The calculation of $\Delta E_{\text{Competitor}}$ was based on the experimental complex structure of the protein and the natural ligand. The calculated binding energy of the query ligand was required to be lower than $\beta \Delta E_{\text{Competitor}}$ for each protein, where $\beta \le 1$. A value of 0.8 for $\beta$ was recommended by the authors for both weak and strong binders.

In addition to the use of a threshold for binding scores obtained from the known protein–ligand complexes, Li *et al.* (2011) introduced consensus scoring to an IVS study. Consensus scoring is a combination of multiple scoring functions. Since every scoring function has its advantages and limitations, consensus scoring provides a way to combine the advantages from different scoring functions. In the work by Li *et al.* two different scoring functions, an empirical scoring function (ICM) and a knowledge-based scoring function (PMF), were employed for consensus scoring, leading to a clear enhancement in hit-rates.

In the web server SePreSA developed by Yang *et al.* (2009), a 2-directional Z-transformation (2DIZ) algorithm was used to process a docking-score matrix. Briefly, 79 proteins with co-crystalized ligands in the target database were selected to dock with 86 ligands, generating a docking-score matrix of 79 × 86 elements. Then, the Z-score was calculated by $Z_{ij} = {{\left( {X_{ij} - \overline{{X_{j} }} } \right)} \mathord{\left/ {\vphantom {{\left( {X_{ij} - \overline{{X_{j} }} } \right)} {{\text{SD}}_{{X_{j} }} }}} \right. \kern-0pt} {{\text{SD}}_{{X_{j} }} }}$, where *X*_*ij*_ is the docking score of ligand *j* to protein *i*, and $\overline{{X_{j} }}$ is the average docking score of ligand *j* against 79 proteins. ${\text{SD}}_{{X_{j} }}$ is the standard deviation of docking scores for ligand *j* with those proteins. The Z-score matrix could be further normalized to a Z′-score matrix, in which the vector for each protein is normalized to a mean of zero and a standard deviation of one. According to results presented in the work, the 2DIZ algorithm significantly improved the prediction accuracy, compared to simply using docking score functions.

Another approach of the normalization of binding energies introduced by Lauro *et al.* (2011) was studying docking of multiple ligands against multiple proteins. The normalization was based on the equation $V = V_{0} /\left[ {\left( {M_{\text{L}} + M_{\text{R}} } \right)/2} \right]$, where $V_{0}$ is the binding energy calculated by the scoring function for each protein–ligand complex, $M_{\text{L}}$ is the average binding energy of each ligand with different proteins, and $M_{\text{R}}$ is the average binding energy of each protein with different ligands. Then, *V* was a normalized value associated with each ligand. The approach effectively avoided the selection of false positive results.

In a recent work by Santiago *et al.* (2012), a selected ligand dataset, the National Cancer Institute (NCI) Diversity Set I containing 1990 drug-like molecules, was used to calibrate binding scores of a query ligand against the proteins in a database. Specifically, the molecules in the NCI Diversity Set I were docked to each protein in the protein database. Then, the top-200, top-20, and Boltzmann-weighted averages of the binding scores were calculated, which served as the references for each protein. If the calculated binding score of the query ligand against a protein was lower than the reference score, the protein was considered as a hit. According to the work, the reference using the top-20 average performed better than the other two averages.

### Web servers

To run an IVS, in addition to the time-consuming and labor-intensive process for the construction of a target database, programming skills and experiences are required to handle hundreds of dockings and to conduct post analysis, which could be tough for researchers focusing on experimental methods. Therefore, several web servers were developed for public use. The only thing that a user would need to do is to provide a small molecule of interest. Then the server automatically runs the IVS and outputs a list of potential targets. Available web servers of docking-based IVS are reported in Table 2.

**Table 2:** Available web servers of the docking-based IVS

Web server	Description	URL
TarFisDock	Using DOCK4.0 as the docking engine and PDTD as the target database. Scores calculated by a force-based scoring function implemented in DOCK4.0 are used for the ranking of targets. Top 2%, 5%, or 10% of the ranking list can be output	http://www.dddc.ac.cn/tarfisdock
SePreSA	Focusing on targets related to SADRs. DOCK4.0 is employed as the docking engine and the database contains 91 SADR proteins. In addition to the scoring function implemented in DOCK, Z-scores are also calculated for the selection of potential targets	http://sepresa.bio-x.cn
DRAR-CPI	Provided by the same groups of SePreSA. The server was designed for drug repositioning by taking ADR into account. DOCK6.0 is employed as the docking engine and the target database contains 353 targetable human proteins. Similar strategy of scoring as in SePreSA is used for the selection of potential targets	http://cpi.bio-x.cn/drar
idTarget	Using MEDock as docking engine and AutoDock4^RAP^ as scoring function. Z-scores calculated based on affinity profiles of binding pockets are used for the selection of potential targets. A “contraction-and-expansion” strategy is used to extend the searching space	http://idtarget.rcas.sinica.edu.tw
DockoMatic	DockoMatic is a local program with GUI. AutoDock and AutoDock Vina can be selected as docking engine. BLAST and MODELER programs are implemented, allowing the user to easily extend the target database based on homology modeling	https://sourceforge.net/projects/dockomatic

Target fishing dock (TarFisDock) (Li *et al.* 2006) is the earliest freely accessible web server using the docking-based IVS technique. In this web server, PDTD is used as the target database, which contains 841 known and potential drug targets. DOCK4.0 (Ewing *et al.* 2001) is employed as the docking engine, and a force field-based scoring function implemented in DOCK is used for binding energy calculation. During docking, ligand flexibility is taken into account, whereas the protein under consideration is treated as rigid. Top 2%, 5%, or 10% of the ranking list can be output for users. Two multi-target ligands, vitamin E (14 known targets) and 4H-tamoxifen (ten known targets), were tested in the study. Top 2% of the ranking list covered 30% of known targets for the two cases. Moreover, 50% of the known targets of vitamin E and 4H-tamoxifen were covered by 10% and 5% of the ranking list, respectively. The TarFisDock server provides a convenient and rapid way to identify potential targets for a given small molecule. Because many of the proteins in PDTD are involved in different therapeutic areas, TarFisDock is a desirable tool for drug repositioning.

SePreSA (Yang *et al.* 2009) is the first docking-based web server focusing on targets related to severe adverse drug reactions (SADRs). The database contains 91 SADR proteins consisting of major phase I and II drug-metabolite enzymes, several human MHC I proteins, and pharmacodynamic proteins. DOCK4.0 is employed as the docking engine. Besides the scoring function implemented in DOCK, the 2DIZ algorithm is applied to generate a Z-score matrix or Z’-score matrix, which calculates the relative ligand–protein interaction strength. In a test of prediction for true and unidentified binding compounds, the value of the area under the curve (AUC) increases from 0.62 (using only the docking-score matrix) to 0.82 (using the 2DIZ algorithm). Therefore, SePreSA is a desirable tool to predict possible side effects of an interesting molecule in the early stage of drug design.

Drug repositioning potential and ADR via chemical–protein interaction (DRAR-CPI) (Luo *et al.* 2011) is another web server provided by the same group who developed SePreSA. The server was designed for drug repositioning by taking ADR into account. The target database contains 353 targetable human proteins with 385 binding sites. Also collected were the information of 254 forms of 166 small molecules with known ADR. Similar to SePreSA, DOCK6.0 (Lang *et al.* 2009) is employed as the docking engine of DRAR–CPI, and the 2DIZ algorithm is applied to generate a Z-score matrix or Z’-score matrix based on docking scores. Furthermore, the server uses an approach to evaluate the drug–drug associations based on gene-expression profiles, searching for similar or opposite drugs from the database for a query ligand. Because the drug–drug association method is beyond this review, the interested reader is recommended to read the original paper (Luo *et al.* 2011).

Recently, Wang *et al.* (2012a) released another docking-based IVS web server named idTarget. The docking engine is maximum-entropy based docking (MEDock) (Chang *et al.* 2005), which was also published as a web server by the same group. AutoDock4^RAP^ (Wang *et al.* 2011), an improved version of the scoring function AutoDock4 (Huey *et al.* 2007), is used for the evaluation of potential targets. The Z-score of a ligand against a protein pocket is calculated based on an affinity profile of the binding pocket (Wang *et al.* 2012a). Then, the ranking of the potential targets for a query ligand is based on their Z values. To screen a large protein structure database, such as the whole PDB database, the authors introduced a “contraction-and-expansion” strategy. In the contraction stage, the target database contains 2091 targets, which were constructed based on sc-PDB. Briefly, 3046 mean points of sc-PDB were clustered with a cutoff of 40% protein sequence identity. In sc-PDB, a mean point is a representative of a cluster containing entries of a protein bound with different ligands. The query ligand is firstly docked to the contracted database, and half of the targets with lower docking energies will be used for the next expansion stage. In the expansion stage, proteins that are homologous or contain similar binding pockets collected from both sc-PDB and PDB are also selected for screening.

In addition to the web servers described above, Bullock *et al.* provided a free and open source program DockoMatic2.0 (Bullock *et al.* 2013), with which the user is able to perform docking-based IVS through a graphical user interface (GUI). AutoDock (Morris *et al.* 1998) or AutoDock Vina (Trott and Olson 2010) can be selected as the docking engine, and the target database is provided by the user. Although the program DockoMatic2.0 is less convenient to use than web servers which only require a user to upload a query ligand, DockoMatic2.0 can be applied to a user-customized target database which is usually not allowed by web servers. It is worthy to note that the basic local alignment search tool (BLAST) (Altschul *et al.* 1997) and MODELER program (Sali and Blundell 1993) are also implemented in DockoMatic2.0. Thus, a user can extend the target database based on homology modeling.

## Applications

### Target identification

Natural products have become an abundant resource for new drug discovery, due to the accumulation of ancient medical knowledge for thousands of years (Ji *et al.* 2009). Identification of the targets for these natural products can not only demystify traditional medicines, but also provide meaningful targets for modern drug design. There are a number of successful stories that utilize docking-based IVS to assist in identifying targets for natural ligands. Do *et al.* used an in-house developed strategy named Selnergy (Do and Bernard 2004), which is based on using the FlexX docking program (Rarey *et al.* 1996) to identify targets for two natural products, ε-viniferin (Do *et al.* 2005) and meranzin (Do *et al.* 2007). From a manually collected database containing 400 targets, cyclic nucleotide phosphodiesterase 4 (PDE4) was identified as a target of ε-viniferin, and three targets, COX1, COX2, and PPARγ, were identified as the targets of meranzin. Lauro *et al.* applied the IVS method to a set of ten phenolic natural compounds (Lauro *et al.* 2012). The target database consists of 163 proteins that are involved in the cancer process. The AutoDock Vina program was employed as the docking engine and the binding energies were normalized to rank the targets. Protein kinases PDK1 and PKC were confirmed as the targets of xanthohumol and isoxanthohumol through *in vitro* biological tests. Recently, the method became popular in the studies of traditional Chinese medicine (TCM) (Yue *et al.* 2008; Feng *et al.* 2011; Chen and Ren 2014). In the study by Chen and Ren (2014), the idTarget server (Wang *et al.* 2012a) along with a ligand-based IVS server PharmMapper (Liu *et al.* 2010b) was employed to identify the potential anticancer targets of Danshensu, an active compound from a widely used TCM Danshen (*Salvia miltiorrhiza*). The screening proposed GTPase HRas as a potential target of Danshensu for further study.

Toledo-Sherman *et al.* (Slon-Usakiewicz *et al.* 2004; Toledo-Sherman *et al.* 2004) developed a chemical proteomics approach, combining (experimental) ultra-sensitive mass spectrometry with (computational) docking-based IVS. This proteomics approach was applied to the exploration of the action mechanism of methotrexate (MTX), an important drug used in cancer, immunosuppression, rheumatoid arthritis, and other highly proliferative diseases. Besides the three main known targets dihydrofolate reductase, thymidylate synthetase, and glycinamide ribonucleotide transformylase, at least eight other proteins were identified as the potential targets of MTX. By using a frontal affinity chromatography with mass spectrometry detection, the authors further confirmed one of these predicted targets, hypoxanthine–guanine amidophosphoribosyltransferase (HGPRT), as a real binder of MTX with a *K*_d_ of 4.2 μmol/L.

In another early application, Muller *et al.* applied IVS to searching for protein targets for a novel chemotype that uses five representative molecules from a combinatorial library that share a 1,3,5-triazepan-2,6-dione scaffold (Muller *et al.* 2006). A collection of 2148 binding sites (Release 1.0 of the sc-PDB (Kellenberger *et al.* 2006)) extracted from the PDB database was screened by the GOLD 2.1 docking program (Jones *et al.* 1997). Five proteins were selected from the top 2% scoring targets by some customized criteria for further experimental evaluation. Two secreted phospholipase A2 isoforms were successfully identified as the real targets of 1,3,5-triazepan-2,6-diones.

Moreover, high throughput screening (HTS) can quickly screen for potential drug candidates; however, the action mechanisms of the resulting candidates are elusive and further improvement of the potency is therefore difficult. IVS can be used to identify the potential targets of these compounds. An example is PRIMA-1 (p53 reactivation and induction of massive apoptosis). PRIMA-1 has the ability to restore the tumor suppressor function of mutant p53, leading to apoptosis in several types of cancer cells. Our group (Grinter *et al.* 2011) used MDock (Huang and Zou 2007a; Yan and Zou 2016) as the docking engine and ITScore (Huang and Zou 2006a, b) as the scoring function to screen the PDTD target database (Gao *et al.* 2008). The highest ranked human protein oxidosqualene cyclase (OSC) was suggested to be the primary binding target of PRIMA-1 and a novel anticancer therapeutic target.

Besides the wide applications in the drug design pipeline, IVS is applied to other fields such as environmental engineering and biosafety of nanomaterials. For example, Xu *et al.* has applied IVS to identifying the potential targets of persistent organic pollutants (POPs) such as dichlorodiphenyldichloroethylene (4,4′-DDE) and polychlorinated biphenyls (PCBs) (Xu *et al.* 2013). The toxicity mechanism of these POPs could be further illustrated. Calvaresi and Zerbetto have also used IVS to identify the protein targets of nanoparticle fullerene C_60_ (Calvaresi and Zerbetto 2010).

### Side effects and toxicity

Side effects and toxicity are mainly responsible for the failure of the compounds in clinical trials, and also for the restricted use or withdrawal of approved drugs. Therefore, taking side effects into account in the initial step of new drug design could significantly increase the final success rate of drug development and drug safety.

Chen *et al.* first tested their in-house, docking-based IVS program named INVDOCK (Chen and Zhi 2001), on the side effects and toxicity of eight clinical agents, aspirin, gentamicin, ibuprofen, indinavir, neomycin, penicillin G, 4H-tamoxifen, and vitamin C (Chen and Ung 2001). It was found that 83% of the experimentally known side effects and toxicity targets could be predicted. Lately, the authors applied the approach to 11 marketed anti-HIV drugs, including protease, nucleoside reverse transcriptase, and non-nucleoside reverse transcriptase inhibitors (Ji *et al.* 2006). The results showed that over 86% of the adverse drug reactions predicted by INVDOCK were consistent with the adverse reactions reported in literature. The agreement between the predicted results and the experimental data was also achieved in the work of Rockey and Elcock’s (Rockey and Elcock 2002), in which three clinically relevant inhibitors (Gleevec, purvalanol A, and hymenialdisine) were analyzed against a set of protein kinase targets (76 GDP receptors and 113 ADP receptors) by the AutoDock program (Morris *et al.* 1998). The success of these pioneering studies brings confidence to the use of a docking-based IVS approach in practice.

Recently, Ma *et al.* (2011) used INVDOCK to investigate potential toxicity mechanisms of melamine, which was found in infant formula and is responsible for the outbreak of nephrolithiasis among children in China. Four target proteins (glutathione peroxidase 1, beta-hexosaminidase subunit beta, l-lactate dehydrogenase, and lysozyme C) were suggested to be related to nephrotoxicity induced by melamine and its metabolite cyanuric acid. In addition, the authors also found three target proteins (superoxide dismutase, glucose-6-phosphate 1-dehydrogenase, glutathione reductase) that were related to lung toxicity. Furthermore, a biological signal cascade network was constructed based on these predicted target proteins. However, the results need to be verified experimentally.

The IVS approach has also been applied to clozapine, one of the most effective medications for the treatment of schizophrenia. The usage of clozapine is limited by its life-threatening adverse drug reaction (ADR), mainly agranulocytosis. Yang *et al.* (2011) used an IVS approach via the DRAR-CPI server to investigate the ADR across a panel of human proteins (381 unique human proteins with 410 binding pockets) for clozapine. As a reference, olanzapine, an analog of clozapine which has a much lower incidence of agranulocytosis, was also analyzed. With the hypothesis that targets related to agranulocytosis tend to bind clozapine but not olanzapine, HSPA1A (the gene of Hsp70) was identified as the off-target of clozapine. The result was confirmed by the comparison of mRNA expression studies on HSPA1A-related genes inside a leukemia cell line with and without the clozapine treatment.

### Drug repositioning

As aforementioned, even officially approved drugs sometimes bind to off-targets and cause side effects. If the off-target of an approved drug happens to be the therapeutic target for another disease, the drug has a chance for a new use, namely drug repositioning. There are a number of repositioned drugs in the market. For example, sildenafil was primarily developed for angina but later approved for erectile dysfunction. Thalidomide was initially marketed for morning sickness but was later approved for leprosy and also for multiple myeloma. More examples can be found in a review by Ashburn and Thor (2004). Although docking-based IVS seems to be a tailor-made tool for drug repositioning, there have been few successful stories until now.

Recently, Li *et al.* (2011) performed a large-scale molecular docking of small-molecule drugs against protein drug targets, in order to find novel targets for the existing drugs. The drugs and targets in the study were based on the data deposited in the DrugBank 2.5 database (Wishart *et al.* 2006). Overall, 252 human protein drug targets and 4621 approved and experimental small-molecule drugs were collected. The ICM program (Abagyan *et al.* 1994) was employed as the docking engine. The large-scale cross dockings (4621 ligands against 252 receptors) were run on a powerful computer cluster with 1000 processors. A consensus score, consisting of an empirical scoring function ICM (Abagyan *et al.* 1994) and a knowledge-based scoring function PMF (Muegge and Martin 1999; Muegge 2006), was used to evaluate the docking poses. The consensus score performed much better than either the ICM score or the PMF score alone, with the percentage of the known interactions in the prediction set improved from 1.1% (ICM score) or 2.0% (PMF score) to 10.3%. Furthermore, by combining with the ranks of the proteins and drugs, the percentage value for the consensus score reached up to 48.8%, giving the confidence that the other 51.2% proteins were indeed novel targets. Successfully, the cancer drug nilotinib was further confirmed as a potent inhibitor of MAPK14 (*IC*_50_ = 40 nmol/L) by biological tests. MAPK14, also known as p38 alpha, is a target in inflammation, suggesting that nilotinib has a chance for being repurposed for the treatment of rheumatoid arthritis.

### Multi-target therapy/drug–target network

In novel drug design, compounds are usually engineered to bind to a specific target, with the assumption that one drug binds to one target to treat one condition. However, this assumption is now in question, with the high failure rate during the late stage of clinical trials due to efficacy and clinical safety problems (Xie *et al.* 2011) being the main source of the scrutiny. Recent studies suggest that each existing drug binds to, on average, about six target proteins (Azzaoui *et al.* 2007; Mestres *et al.* 2008) instead of one. This phenomenon can be easily understood in a biological network, in which each node represents a protein and a link between two proteins means a direct interaction. Considering the robustness of biological systems, acting on multiple nodes should, in theory, be more effective in affecting the system overall than when only considering one node. Therefore, a multi-target therapy is expected to be able to break the bottleneck of current single-target drug design paradigms. However, the development of multi-target drugs proceeds slowly, partially due to the lack of experimental tools to identify targets on a proteome-wide scale (Xie *et al.* 2011). Thus, computational approaches, such as IVS described in this review, were developed to narrow down the targets of interest for further experimental validation.

An example of docking-based IVS for multi-target identification can be found in a recent work by Zhao *et al.* (2012). The INVDOCK program (Chen and Zhi 2001) was employed to search potential protein targets for astragaloside-IV (AGS-IV). The AGS-IV is one of the main active ingredients of *Astragalus membranaceu*s Bunge, a traditional Chinese medicine for cardiovascular diseases (CVD). The protein targets of approved small-molecule drugs for CVD deposited in the DrugBank database (Wishart *et al.* 2006) were collected as the target database, consisting of 188 proteins. Among the 39 predicted targets, three proteins (calcineurin, angiotensin-converting enzyme, and c-Jun N-terminal kinase) were experimentally validated at a molecular level. By mapping the 39 proteins onto the protein–protein interaction network of the human genome, 34 of them can be linked into a sub-network, which can be further divided into six topologically compact modules. The effects of AGS-IV on CVD were supposed to act through binding to multiple targets, for example, by directly binding to the hubs of six modules. The results were further confirmed by the comparison with the drug–target networks of the approved CVD drugs that share common targets with AGS-IV.

### Receptor design

In addition, the docking-based IVS method could be used for receptor design. Steffen *et al.* (2007) successfully improved the property of a synthetic receptor for a binding ligand. In this study, camptothecin (CPT) was chosen as the investigated ligand. Although CPT presents remarkable anticancer activity in preliminary clinical trials, its therapeutic potential is hampered by its low solubility and stability. Thus, hosts or so-called receptors were designed for the solubilization of the ligand. In particular, a set of β-cyclodextrin (β-CD) derivatives (a total of 1846 entities) was generated from the β-CD core and thiol building blocks as the receptor candidates (from the target database). CPT was docked to each β-CD derivative in the target database by two different docking programs, AutoDock 3.05 (Morris *et al.* 1998) and GlamDock 1.0 (Tietze and Apostolakis 2007). Nine receptors from the top 10% candidates were selected for experimental validation. Successfully, five of them significantly improved the solubility of CPT, and their ability to do so was significantly better than any other known CD derivative.

## Challenges

In summary, during the last decade, the entire field of docking-based IVS, including the construction of target databases, scoring functions, and post analysis, has been significantly improved by researchers from all over the world. A number of successful applications as described in this review have proved that docking-based IVS is a powerful technique for drug discovery. However, several challenges remain to be solved for docking-based IVS to become a robust tool.

The first challenge is the incompleteness of available target databases. Using the data in DrugPort (http://www.ebi.ac.uk/thornton-srv/databases/drugport/) as an example, there are a total of 1664 known druggable protein targets in the database, but only about half of them have 3D structures in the PDB. If unknown targets are considered, this rate could be much lower. Furthermore, these targets with known-structures are not evenly distributed among different superfamilies, due to experimental limitations. For example, the superfamily of membrane proteins, the G-protein-coupled receptors (GPCRs), is one of the most important targets in drug design, given the fact that they account for over a quarter of the known drug targets (Overington *et al.* 2006), and about half of the drugs on the market target GPCRs specifically (Klabunde and Hessler 2002). However, only a fraction of the GPCRs have experimental structures (Venkatakrishnan *et al.* 2013), because the structural resolution of membrane proteins like GPCRs is much more complicated and difficult to elucidate than global proteins such as enzymes. Fortunately, the current databases can be significantly improved through homology modeling techniques, and the incompleteness problem can be gradually solved with time as more and more complete structures are determined by experimental methods.

Another challenge is from the vantage point of protein flexibility. As aforementioned, protein–ligand binding is a mutual fitting process. The existing docking programs are able to account for the flexibility of small molecules very well, but the overall flexibility of the entire protein remains a great challenge. Efforts have been made to partially consider protein flexibility during docking. For example, the side chains of the residues in the active site can be treated to be flexible with the induced-fit docking strategies (Sherman *et al.* 2006). In another example, an ensemble of protein structures are used for docking in MDOCK (Huang and Zou 2007a, b). However, flexible docking using the induced-fit strategy is time-consuming. For the ensemble docking using MDOCK, an ensemble of experimentally determined protein structures are not always available. These methods are usually difficult to be directly applied to IVS studies which involve hundreds of different proteins. To the best of our knowledge, the proteins were all treated as rigid bodies in the published docking-based IVS studies. Thus, it would be useful to develop efficient protein flexibility algorithms for IVS studies.

At this stage, IVS and the more traditional VS work as an enrichment method rather than an accurate prediction tool, mainly due to the inaccuracy of the scoring functions. Simply selecting the top targets in the ranking list could result in many false positive candidates. As reviewed in the subsection on scoring functions, efforts have been made to improve the success rate, including setting a threshold for each target, using consensus scoring functions, or normalizing binding scores. However, all these methods can be regarded as post analysis, which are highly dependent on the scoring values calculated by the existing inaccurate scoring functions. In fact, the scoring function could be the biggest challenge for molecular docking. A detailed review about scoring functions for protein–ligand docking can be found in a recent review (Huang *et al.* 2010). Recently, Wang *et al.* (2012b) evaluated the performance of Glide scoring functions in IVS based on the Astex diverse set. Interestingly, “interprotein noises” were found in the Glide scores, suggesting that scoring functions that are developed for conformational (the same complex) ranking could result in over- or underestimated scores when they are directly used for the ranking of different protein–ligand complexes. By introducing a correction term based on a given protein characteristic, the ratio of the relative hydrophobic and hydrophilic character of the binding site, the accuracy of target prediction was improved by 27% (*i.e.*, from 57% to 72%). The study could be used as a reference in the optimization of the existing scoring functions for IVS studies.

An efficient way to address the above challenges (*i.e.*, protein flexibility and scoring function) could be the use of more accurate yet more time-consuming sampling/scoring strategies for the enriched subset (*e.g.*, top 5% of the targets). Regarding the sampling aspect, protein flexibility could be partially considered by using ensemble docking or induced-fit docking strategies. Regarding the scoring aspect, contributions from the solvent effect and from the conformational entropic effect could be considered. Well-studied strategies are molecular dynamics (MD)-based binding free energy calculation methods, such as MM/PBSA and MM/GBSA (Srinivasan *et al.* 1998; Kollman *et al.* 2000; Wang *et al.* 2001). In addition, recent studies show that polarization effects are important for both binding mode and binding affinity predictions (Cho *et al.* 2005; Xu and Lill 2013). To efficiently consider polarization effects in the docking process, quantum mechanics (QM) or hybrid quantum mechanics/molecular mechanics (QM/MM) methods need to be employed. A QM-polarized ligand docking method has been implemented in a commercial software package, Schrödinger Suites (https://www.schrodinger.com).

There are many docking programs and scoring functions that can be used for an IVS study. As reviewed in this paper, some of them have already been used by different groups for different purposes with varying degrees of success. It would be interesting to find which programs are more effective for IVS studies than others. Such an attempt has been tried by Liu *et al.* (2010a). In their work, five schemes, GOLD (Jones *et al.* 1997) and FlexX (Rarey *et al.* 1996) implemented in Sybyl, TarFisDock (Li *et al.* 2006) which is based on DOCK4.0 (Ewing *et al.* 2001), and two in-house docking strategies, TarSearch-X and TarSearch-M (DOCK5.1 (Moustakas *et al.* 2006)) combined with two in-house scoring functions X-Score (Wang *et al.* 2002) and M-score (Yang *et al.* 2006), were tested for eight multi-target compounds extracted from DrugBank (Wishart *et al.* 2006). The target database was collected from the PDB, and contained 1714 entries from 1594 known drug targets. According to the order of the known targets in the rank list, their results show that TarSearch-X is the most efficient and GOLD is acceptable. However, the study has some limitations. Seven of the eight selected multi-target compounds have only two known targets. Another compound has three known targets. More convincing validation would be to use compounds that have many known targets, such as vitamin E with 14 known targets and 4H-tamoxifen with ten known targets which were used in the test for TarFisDock (Li *et al.* 2006). In addition, a number of other powerful docking programs and scoring functions are awaited to be assessed for IVS studies.

To effectively evaluate a method of docking-based IVS, a database is desired to contain both positive and negative results. However, negative data are difficult to collect because literature prefer to present successful cases rather than failed cases, *i.e.*, in which a molecule does not interact with a protein. Fortunately, Schomburg and Rarey (2014) recently provided an example of such a database. Because of the limited data available for negative results, the authors constructed a small set with both positive and negative results. This small set, referred to as the selectivity dataset, consists of a total of eight proteins belonging to three target classes and 17 small molecules with defined selectivity in the respective target class. The selectivity dataset is suggested to be used for proof-of-concept studies. A large dataset containing 7992 protein structures and 72 drug-like ligands was also provided. The dataset, called Drugs/sc-PDB dataset, was constructed based on the data in DrugBank (Wishart *et al.* 2006) and sc-PDB (Kellenberger *et al.* 2006). The 72 drug-like ligands were selected based on the assumption that the selectivity and targets of the approved drugs have been well studied. The selectivity dataset and the Drugs/sc-PDB dataset form a benchmark for target identification methods.

The last challenge could potentially be the post-analysis problem. The output of IVS is an enriched subset, which contains at least tens of potential targets (including false positive targets). How to connect these predicted multiple targets to the mechanisms of the ligand remains an open question. Usually, the predicted targets need to be validated by biological experiments. Only then can biological functions of the true targets be connected to the phenotypic effects of the ligand. Recently, the biological network idea was employed for the analysis of IVS results. In the work by Zhao *et al.* (2012), predicted targets were mapped onto the protein–protein interaction network of the human genome. A sub-network was identified that could effectively explain a connection to the actual mechanisms of the ligand in question.
